# Comparing the Efficacy and Safety of Glucagon-Like Peptide 1 Receptor Agonists with Sodium-Glucose Cotransporter 2 Inhibitors for Obese Type 2 Diabetes Patients Uncontrolled on Metformin: A Systematic Review and Meta-Analysis of Randomized Clinical Trials

**DOI:** 10.1155/2020/1626484

**Published:** 2020-09-28

**Authors:** Lu Ding, Bang Sun, Xinhua Xiao

**Affiliations:** Department of Endocrinology, Key Laboratory of Endocrinology, Ministry of Health, Peking Union Medical College Hospital, Diabetes Research Center of Chinese Academy of Medical Sciences and Peking Union Medical College, Beijing 100730, China

## Abstract

**Introduction:**

To conduct the first meta-analysis of randomized controlled trials (RCTs) comparing glucagon-like peptide 1 receptor agonists (GLP-1RAs) with sodium-glucose cotransporter 2 inhibitors (SGLT-2is) for obese type 2 diabetes (T2D) patients uncontrolled on metformin.

**Methods:**

We searched Pubmed, Embase, Cochrane Central Register of Controlled Trials (CENTRAL), Ovid, and Web of Science from inception to May 14, 2020, without language restrictions for eligible RCTs. The primary outcome is the mean change from baseline in glycated haemoglobin (HbA1c).

**Results:**

Totally, 3 RCTs enrolled 2066 patients were identified. Compared with SGLT-2is, treatment with GLP-1RAs achieved significant reduced HbA1c by 0.40% (95% CI: −0.54, −0.25; *p* < 0.00001), fasting blood glucose (FBG) by 0.17 mmol/L (95% CI: −0.31, −0.04; *p*=0.01), and postprandial blood glucose (PBG) by 0.32 mmol/L (95% CI: −0.49, −0.14; *p*=0.0003) for obese T2D patients uncontrolled on metformin. The significant benefit of weight loss was seen in semaglutide (MD: −0.75; 95% CI: −1.18, −0.31; *p* < 0.0007). No significant difference was detected between GLP-1RAs and SGLT-2is in overall adverse events (RR: 1.03; 95% CI: 0.98, 1.09; *p*=0.76), but gastrointestinal events showed higher occurrence in GLP-1RAs groups compared with SGLT-2is (RR: 1.62; 95% CI: 1.37, 1.93; *p* < 0.00001). Subgroup analyses revealed that follow-up time did not statistically influence glycemic control.

**Conclusion:**

GLP-1RAs are superior to SGLT-2is for obese T2D patients uncontrolled on metformin in glycemic control without an increase in adverse events except for a higher occurrence in gastrointestinal events. Future large longer-term follow-up clinical trials are needed to provide more evidence about the sustainable effects and safety of GLP-1RAs compared with SGLT-2is.

## 1. Introduction

Type 2 diabetes (T2D), the most common type of diabetes, is recognized as the “disaster of the 21st century” by the World Health Organization (WHO) [1, 2]. Generally, lifestyle modification and metformin monotherapy are recommended in the first line for T2D patients, but lots of patients still experience unsatisfactory glycemic control due to progressive deterioration of T2D [3, 4]. Additional hypoglycemic agents are required to start a combination therapy under such circumstance [5]. Therefore, new medications are developed and made available constantly.

Among all the pharmacologic therapies available for second-line treatment for T2D, glucagon-like peptide 1 receptor agonists (GLP-1RAs) and sodium-glucose cotransporter 2 inhibitors (SGLT-2is) are particularly gaining popularity owing to their well-established hypoglycemic efficacy, significant remission rate of complications, and reliable benefits such as weight loss [3, 6]. GLP-1RAs are categorized as short-acting and long-acting compounds. The short-acting GLP-1RAs predominantly lower postprandial blood glucose (PBG) through inhibition of gastric emptying, whereas the long-acting GLP-1RAs reduce blood glucose mainly through stimulation of insulin secretion, which is more effective in glycemic control [7, 8]. SGLT-2is are a class of oral antidiabetic drugs which facilitate glycosuria by selectively inhibiting renal reabsorption of glucose and sodium in the proximal tubule [9, 10]. The American Diabetes Association (ADA) recommended a patient-centered approach to guide the selection of pharmacologic agents and gave preference to a GLP-1RA or an SGLT-2i when metformin is not sufficient in meeting one's glycemic targets [11]. Current evidences make the treatment option clear in T2D patients with established cardiovascular disease (CVD) or chronic kidney disease (CKD) [6, 12]; however, it remains obscure which one is better for obese T2D patients uncontrolled on metformin.

To the best of our knowledge, although network meta-analyses comparing GLP-1RAs with SGLT-2is were performed, the nature of indirect comparison contributes to excessive biases to the outcomes [13–15]. In 2019, two head-to-head randomized controlled trials (RCTs) comparing these two classes of agents were currently available [16, 17]. Thus, this study aims to conduct a systematic review and meta-analysis of RCTs to compare the efficacy and safety outcomes of GLP-1RAs and SGLT-2is for obese T2D patients uncontrolled on metformin.

## 2. Methods

This systematic review and meta-analysis followed prior determined inclusion criteria. The study was performed under the guidance of the Cochrane Handbook for Systematic Reviews of Interventions [18] and was reported according to the PRISMA Statement [19].

### 2.1. Data Sources and Searches

Systematically electronic search was performed in PubMed, Embase, Cochrane Central Register of Controlled Trials (CENTRAL), Ovid, and Web of Science from inception to 14 May 2020 to identify eligible RCTs without restrictions on language. An additional search was carried out in ClinicalTrials.gov, and the reference lists of included potentially relevant studies were examined manually to identify any additional studies. The details of the search strategy were shown in Table S1.

### 2.2. Study Selection

RCTs were considered eligible for inclusion if they (1) recruited T2D patients who showed inadequate response to stable and optimized metformin monotherapy, with a body mass index ≥30 kg/m^2^, (2) compared the efficacy and safety outcomes of GLP-1RAs with SGLT-2is directly, (3) reported the mean change in glycated haemoglobin (HbA1c) from baseline, and (4) had a follow-up time for at least 12 weeks. Duplicate reports and RCTs that did not meet the inclusion criteria were excluded. If more than one article were published on an overlapping population, the most comprehensive article was included in the meta-analysis. Two investigators completed the selection process independently. Discrepancies were resolved by consensus discussions.

### 2.3. Data Extraction and Quality Assessment

The following information was extracted from each included trial: the first author, year of publication, sample size, intervention drugs and dosages, follow-up duration, baseline participant characteristics including age, gender, anthropometrics, and duration of T2D. The primary outcome was the mean change from baseline in HbA1c. Secondary outcomes included the mean change from baseline in fasting blood glucose (FBG), PBG, and bodyweight. Safety outcomes were overall adverse events (AEs) and AEs of specific interest including hypoglycemia, urinary tract infections, and gastrointestinal events. Data were independently extracted by the author (Lu Ding) using standard data extraction forms, and another author (Bang Sun) examined the extracted data for accuracy. Any disagreements were resolved by consensus discussions or adjudicated by the third reviewer when necessary. Two authors independently assessed the risk of selection, detection, performance, reporting, and attrition bias of RCTs with Cochrane Collaboration's tool [18]. The quality of evidences was assessed by the grading of recommendations assessment, development, and evaluation (GRADE) methodology [20].

### 2.4. Data Synthesis and Analysis

All statistical analyses were performed with Review Manager (RevMan, version 5.3) with a *p* < 0.05 considered statistically significant. The random-effect model was used for synthesizing all outcomes. For continuous and dichotomous data, differences were analyzed by mean differences (MDs) and relative risks (RRs), respectively, with 95% confidence intervals (CIs). Statistical heterogeneity was quantified mainly by the *I*^2^ statistic, with *I*^2^ values greater than 50% proving high heterogeneity. Possible publication bias was evaluated by funnel plots when more than 10 studies were included. Subgroup analyses were performed in efficacy outcomes. Moreover, sensitivity analysis was performed for the primary outcome by excluding one trial at a time to make sure the stability of the results.

## 3. Results

### 3.1. Study Characteristics

**Figure 1:**
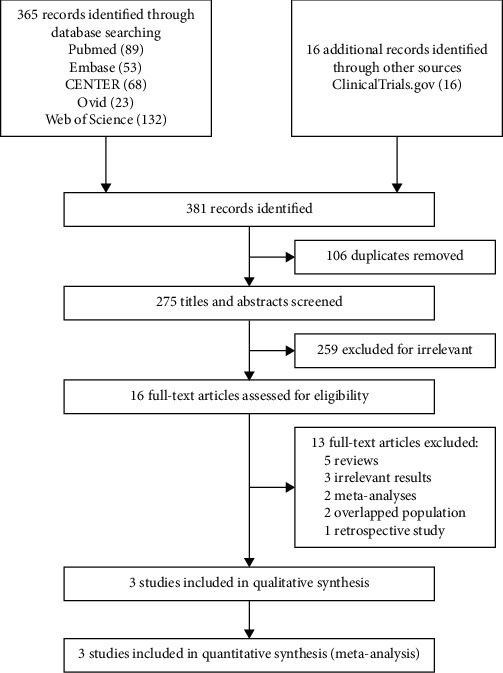
Flow diagram of study selection.

An overview of the search process was shown in Figure 1. Of 381 identified articles, 3 RCTs enrolling 2066 obese T2D patients were eligible for inclusion for this meta-analysis [16, 17, 21]. The characteristics of trails and baseline of the enrolled participants were presented in Table 1.

To sum up, the mean age of participants (range from 54 to 58 years), the proportion of women (range from 43% to 52%), and the mean length of diabetes (range from 7.1 to 7.7 years) were similar across three RCTs. The follow-up time ranged from 26 to 52 weeks. And the mean baseline HbA1c ranged from 8.1 to 9.3%. Of all the 2066 participants, 48.5% were female and most of them were white. Two classes of long-acting GLP-1RAs were evaluated: semaglutide (orally 14 mg/week, subcutaneously 1 mg/week) and exenatide (subcutaneously 2 mg/week), and three classes of orally SGLT-2is were examined: empagliflozin (25 mg/day), canagliflozin (300 mg/day), and dapagliflozin (10 mg/day).

The quality assessment of RCTs was summarized in Table S2. Two of the RCTs reported low risk of bias, while one of the RCTs showed performance bias due to lack of blinding. The publication bias has not been assessed because less than 10 RCTs were included in this meta-analysis. However, since a limited number of RCTs were included and all of them were sponsored by companies, publication bias was strongly suspected.

### 3.2. Glycemic Control

**Figure 2:**
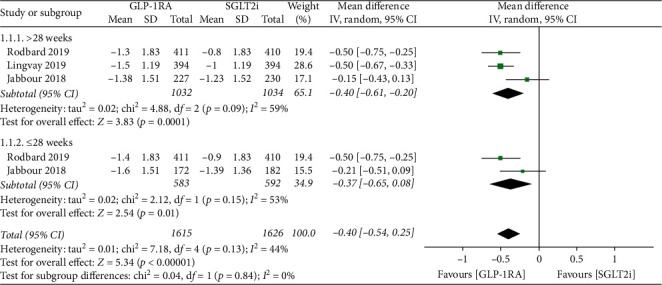
Forest plot for meta-analyses comparing the effect of GLP-1RAs with SGLT-2is in HbA1c. GLP-1RAs: glucagon-like peptide 1 receptor agonists, SGLT-2is: sodium-glucose cotransporter 2 inhibitors, HbA1c: glycated haemoglobin, CI: confidence interval, SD: standard deviation.

Overall, polled results showed that GLP-1RAs significantly reduced HbA1c by 0.40% (95% CI: −0.54, −0.25; *p* < 0.00001) compared with SGLT-2is (Figure 2). On account of one of the RCTs lacking blinding and strongly suspected publication bias, the quality of this evidence was low according to GRADE (Table 2). Sensitivity analysis showed that no matter which study was removed, the results revealed were similar to those of the main analysis (Table S3).

FBG and PBG were also detected, and both of them showed significant decreases in GLP-1RAs treatment compared with SGLT-2is (Figures S1 and S2). GLP-1RAs lowered FBG by 0.17 mmol/L (95% CI: −0.31, −0.04; *p*=0.01), and reduced PBG by 0.32 mmol/L (95% CI: −0.49, −0.14; *p*=0.0003) compared with SGLT-2is.

### 3.3. Body Weight

**Figure 3:**
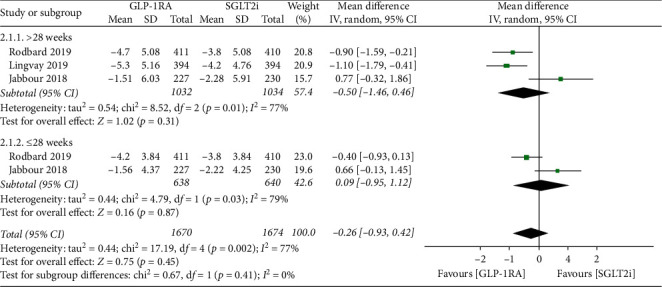
Forest plot for meta-analyses comparing the effect of GLP-1RAs with SGLT-2is in bodyweight. GLP-1RAs: glucagon-like peptide 1 receptor agonists, SGLT-2is: sodium-glucose cotransporter 2 inhibitors, CI: confidence interval, SD: standard deviation.

Pooled results of the mean change of bodyweight from baseline showed no significant difference between the treatment of GLP-1RAs and SGLT-2is (MD: −0.26; 95% CI: −0.93, 0.42; *p*=0.45) with a substantial heterogeneity (*I*^2^ = 77%) (Figure 3). However, restricting the analysis to the class of semaglutide of GLP-1RAs revealed a significant reduction of bodyweight by 0.75 kg (95% CI: −1.18, −0.31; *p*=0.0007) compared with SGLT-2is (Figure S3).

### 3.4. Adverse Events

**Figure 4:**
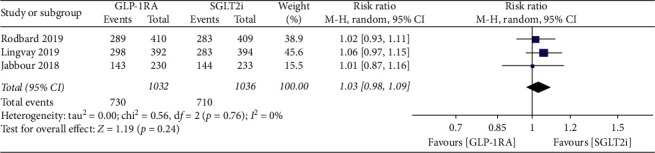
Forest plot for meta-analyses comparing the overall safety of GLP-1RAs with SGLT-2is. AEs leading to discontinuation includes injury, angioedema and infections, etc. AEs: adverse events, GLP-1RAs: glucagon-like peptide 1 receptor agonists, SGLT-2is: sodium-glucose cotransporter 2 inhibitors, CI: confidence interval.

Totally, 2 patients died in the treatment progress of GLP-1RAs, and 2 patients died in the treatment progress of SGLT-2is. The overall occurrence of AEs was not significantly different between the treatment of GLP-1RAs and SGLT-2is (RR: 1.03; 95% CI: 0.98, 1.09; *p*=0.76) (Figure 4).

Some AEs of specific interest were also detected. No statistically significant differences were found in the occurrence of hypoglycemia (RR: 1.38; 95% CI: 0.96, 1.98; *p*=0.08) and urinary tract infections (RR: 0.98; 95% CI: 0.60, 1.58; *p*=0.92) between GLP-1RAs and SGLT-2is group, while the occurrence of gastrointestinal events was higher in GLP-1RAs compared with that in SGLT-2is (RR: 1.62; 95% CI: 1.37, 1.93; *p* < 0.00001) (Figure S4).

### 3.5. Subgroup Analyses

Subgroup analyses were conducted for HbA1c, FBG, PBG, and bodyweight. The mean change from baseline with different follow-up time (>28 weeks and 28 weeks) was assessed, and results did not statistically change with the variable factors.

## 4. Discussion

This systematic review and meta-analysis found that treatment with GLP-1RAs achieved more effective improvements in glycemic control for obese T2D patients uncontrolled on metformin, compared with treatment with SGLT-2is. The significant benefit of weight loss was shown in the class of semaglutide of GLP-1RAs. No significant difference was detected between the two classes of medications in overall safety outcomes, but gastrointestinal events showed higher occurrence when treated with GLP-1RAs compared with SGLT-2is. Subgroup analyses revealed that follow-up time did not statistically influence the treatment effect of glycemic control and weight loss.

The ADA recently updated recommendation for the management of T2D mainly concerning add-on therapy. When the first-line therapy of metformin fails to be effective in glycemic control, it should be determined that whether the arteriosclerotic cardiovascular disease (ASCVD) is present, and if not present, the recommendation gives preference to a GLP-1RA or an SGLT-2i for obese T2D patients [11, 22]. Previous network meta-analysis demonstrated that GLP-1RAs showed superiority in achieving improvements in glycemic control in T2D patients [13, 14], which are consistent with our finding. However, the main limitation of the network meta-analysis is the nature of lacking direct comparisons, which may lead to excessive biases [23]. Our study synthesized data from head-to-head RCTs and achieved direct comparison to avoid systematic differences of the baseline characteristics of participants in different intervention groups, which is quite different from the previous study in terms of methodology. Besides, our study rated the quality of the evidence by GRADE, contributing to a higher credibility of findings compared with the previous studies. This meta-analysis included three recent head-to-head RCTs comparing GLP-1RAs with SGLT-2is, and to the best of our knowledge, it is the first meta-analysis included head-to-head RCTs comparing the efficacy and safety of GLP-1RAs and SGLT-2is in obese T2D patients uncontrolled on metformin.

In this study, results showed that GLP-1RAs significantly reduced HbA1c compared with SGLT-2is regardless of the duration of follow-up time. Because one of the RCTs was open-label and lacked blinding [16] and all the trials was sponsored by companies thus leading to strongly suspected publication bias, the quality of the evidence was low. Luckily, sensitivity analysis proved the stability of the results. FBG and PBG also showed significant decreases in GLP-1RAs group, suggesting that GLP-1RAs achieved effective glycemic control through regulating both of FBG and PBG. The mean change of bodyweight from baseline showed no significant difference between GLP-1RAs and SGLT-2is groups with a substantial heterogeneity. Considering SUSTAIN 3 demonstrated that once-weekly semaglutide led to superior improvement in weight loss compared with exenatide extended release [24], we suspected that the substantial heterogeneity may be associated with different classes of GLP-1RAs. Restricting the analysis according to the class of GLP-1RAs proved a significant reduction of the mean change of bodyweight with the treatment of semaglutide compared with SGLT-2is. However, the result should be considered cautiously for the limited number of included trials.

This study demonstrated no significant differences in overall safety outcomes between the treatment of the two classes of medications except for gastrointestinal events. However, it should be still cautious to make interpretations for evidence of safety outcomes, given the limited number of included trials and the limited number of documented AEs. Moreover, the low incidence of fatal AEs and the great individual variations call for longer-term follow-up verifications. Clinicians are supposed to evaluate the balance of benefits and risks of each drug comprehensively and make sure that patients are fully informed of potential risks, especially those who are vulnerable to gastrointestinal disease.

To the best of our knowledge, this study is the first meta-analysis that included head-to-head RCTs to compare the efficacy and safety of GLP-1RAs and SGLT-2is in obese T2D patients uncontrolled on metformin. Importantly, the study rated the quality of the evidence by GRADE, contributing to a higher credibility of the findings. However, the study also has several limitations. First, a limited number of RCTs were included and a limited number of participants were enrolled in the meta-analysis. Simultaneously, both of the GLP-1RAs and SGLT-2is contain several drugs, but only two to three drugs and dosages were included because of the limited data. While these mean that the study should draw a conclusion cautiously, they also provide evidence that additional clinical trials comparing GLP-1RAs and SGLT-2is in T2D patients uncontrolled on metformin are needed. Second, the pooled results of weight loss had a substantial heterogeneity with an *I*^2^ statistic that reaches up to 77%. We restricted the analysis according to the class of GLP-1RAs to reduce the heterogeneity, but considering only two trials were included in the end, the result should be considered cautiously. Third, the limited number of included studies provided modest ability to examine the presence of publication bias. But the publication bias was strongly suspected because all the trials were sponsored by companies. Finally, the follow-up time was not enough to draw a definitive conclusion about the long-term effects and risks. Therefore, longer-term follow-up and real-world researches are needed to provide more evidence about the sustainable effects and safety of GLP-1RAs compared with SGLT-2is.

## 5. Conclusion

To sum up, the study highlighted that GLP-1RAs are superior to SGLT-2is in the treatment for obese T2D patients uncontrolled on metformin in glycemic control without an increase in total AEs except for a higher occurrence in gastrointestinal events. Weight loss benefit was shown in the treatment of semaglutide. Future large longer-term follow-up clinical trials are needed to provide more evidence about the sustainable effects and safety of GLP-1RAs compared with SGLT-2is.

## Figures and Tables

**Table 1:** Characteristics of the trials and baseline of the enrolled participants.

	Rodbard [16]		Lingvay [17]		Jabbour [21]	
	Oral semaglutide 14 mg/w	Oral Empagliflozin 25 mg/d	Subcutaneous semaglutide 1 mg/w	Oral canagliflozin 300 mg/d	Subcutaneous exenatide 2 mg/w	Oral dapagliflozin 10 mg/d
Patients, *n*	411	410	394	394	227	230
Age (years), mean (SD)	57 (10)	58 (10)	55.7 (11.1)	57.5 (10.7)	54 (10)	55 (9)
Female, *n* (%)	205 (49.9)	201 (49.0)	171 (43%)	193 (49%)	111 (49%)	120 (52%)
Black, *n* (%)	26 (6.3)	33 (8.0)	28 (7%)	30 (8%)	27 (12%)	33 (14%)
White, *n* (%)	355 (86.4)	353 (86.1)	297 (75%)	290 (74%)	194 (85%)	189 (82%)
Asian, *n* (%)	28 (6.8)	21 (5.1)	62 (16%)	63 (16%)	1 (<1%)	1 (<1%)
Other, *n* (%)	2 (0.5)	3 (0.7)	7 (2%)	10 (3%)	5 (2%)	7 (3%)
Hispanic ethnic origin, *n* (%)	91 (22.1)	108 (26.3)	156 (40%)	137 (35%)	91 (40%)	85 (37%)
HbA1c (%), mean (SD)	8.1 (0.9)	8.1 (0.9)	8.3 (1.0)	8.2 (1.0)	9.3 (1.1)	9.3 (1.0)
FBG (mmol/L), mean (SD)	9.5 (2.3)	9.7 (2.5)	9.4 (2.7)	9.4 (2.6)	10.7 (2.8)	10.6 (2.6)
PBG(mmol/L), mean (SD)	—	—	2.1 (1.9)	2.2 (1.8)	—	—
Bodyweight (kg), mean (SD)	91.9 (20.5)	91.3 (20.1)	90.6 (22.6)	89.8 (22.6)	89.8 (20.2)	91.1 (19.7)
BMI (kg/m^2^), mean (SD)	32.2 (6.8)	32.5 (6.9)	32.9 (6.3)	32.8 (5.9)	32.0 (5.9)	33.0 (6.1)
Diabetes duration (years), mean (SD)	7.2 (5.8)	7.7 (6.3)	7.5 (5.9)	7.2 (5.4)	7.4 (5.5)	7.1 (5.5)
Follow up (weeks), *n*	26, 52	—	52	—	28, 52	—

HbA1c: glycated haemoglobin, FBG: fasting blood glucose, PBG: postprandial blood glucose, BMI: body mass index.

**Table 2:** Summary of findings and strength of evidence.

Outcome	Studies (patients)	Mean differences/relative effect (95% CI)	*I* ^2^	Certainty of the evidence
HbA1c (%)	3 (2066)	MD: −0.40 (−0.54, −0.25)	44%	Low^1,2^
FBG (mmol/L)	3 (2066)	MD: −0.17 (−0.31, −0.04)	0	Low^1,2^
PBG (mmol/L)	2 (1609)	MD: −0.32 (−0.49, −0.14)	44%	Low^1,2^
Bodyweight (kg)	3 (2066)	MD: −0.26 (−0.93, 0.42)	77%	Very Low^1,2,3^
Adverse events	3 (2068)	RR: 1.03 (0.98, 1.09)	0	Low^1,2^
Hypoglycemia	2 (1605)	RR: 1.38 (0.96, 1.98)	35%	Low^1,2^
Urinary tract infections	2 (1249)	RR: 0.98 (0.60, 1.58)	0	Moderate^2^
Gastrointestinal events	2 (1249)	RR: 1.62 (1.37, 1.93)	0	Moderate^2^

HbA1c: glycated haemoglobin, FBG: fasting blood glucose, PBG: postprandial blood glucose, CI: confidence interval, RR, risk ratio; MD, mean difference. ^1^Study limitations for one of the trail lacked of blinding. ^2^Strongly suspected publication bias for all the trials were sponsored by companies. ^3^Inconsistency for substantial heterogeneity.

## Data Availability

The datasets generated and/or analyzed during the current study are available from the corresponding author on reasonable request.

## Supplementary Materials

Supplementary Materials Table S1: search strategy. Table S2: quality assessment of randomized controlled trials included in the meta-analysis with the Cochrane Risk of Bias Assessment. A indicates low risk, B indicates unclear, and C indicates high risk. Table S3: Sensitivity analyses for meta-analyses comparing the effect of GLP-1RAs with SGLT-2is in HbA1c. MD: mean difference, CI: confidence interval. Figure S1: forest plot for meta-analyses comparing the effect of GLP-1RAs with SGLT-2is in FBG. CI: confidence interval, SD: standard deviation. Figure S2: forest plot for meta-analyses comparing the effect of GLP-1RAs with SGLT-2is in PBG. CI: confidence interval, SD: standard deviation. Figure S3: forest plot for meta-analyses comparing the effect of semaglutide with SGLT-2is in bodyweight. CI: confidence interval, SD: standard deviation. Figure S4: forest plot for meta-analyses comparing AEs of specific interest of GLP-1RAs with SGLT-2is. (A) Hypoglycemia identified according to the ADA classification (<3.9 mmol/L [<70 mg/dL]). (B) Urinary tract infections. (C) Gastrointestinal events. CI: confidence interval.
