# Supplementary material for: Comparing the Efficacy and Safety of Glucagon-Like Peptide 1 Receptor Agonists with Sodium-Glucose Cotransporter 2 Inhibitors for Obese Type 2 Diabetes Patients Uncontrolled on Metformin: A Systematic Review and Meta-Analysis of Randomized Clinical Trials
Source: Int J Endocrinol. 2020 Sep 28;2020:1626484. doi: 10.1155/2020/1626484 (PMC7539133; doi:10.1155/2020/1626484)
Supplement: Supplementary Materials — Table S1: search strategy. Table S2: quality assessment of randomized controlled trials included in the meta-analysis with the Cochrane Risk of Bias Assessment. A indicates low risk, B indicates unclear, and C indicates high risk. Table S3: Sensitivity analyses for meta-analyses comparing the effect of GLP-1RAs with SGLT-2is in HbA1c. MD: mean difference, CI: confidence interval. Figure S1: forest plot for meta-analyses comparing the effect of GLP-1RAs with SGLT-2is in FBG. CI: confidence interval, SD: standard deviation. Figure S2: forest plot for meta-analyses comparing the effect of GLP-1RAs with SGLT-2is in PBG. CI: confidence interval, SD: standard deviation. Figure S3: forest plot for meta-analyses comparing the effect of semaglutide with SGLT-2is in bodyweight. CI: confidence interval, SD: standard deviation. Figure S4: forest plot for meta-analyses comparing AEs of specific interest of GLP-1RAs with SGLT-2is. (A) Hypoglycemia identified according to the ADA classification (<3.9 mmol/L [<70 mg/dL]). (B) Urinary tract infections. (C) Gastrointestinal events. CI: confidence interval. [file 1626484.f1.doc]

**Supplementary files**

**Supplementary Tables**

**Table S1.** Search strategy.

**Table S2.** Quality assessment of randomized controlled trials included in the meta-analysis with the Cochrane Risk of Bias Assessment.

**Table S3.** Sensitivity analyses for meta-analyses comparing the effect of GLP-1RAs with SGLT-2is in HbA1c.

**Supplementary Figures**

**Figure. S1.** Forest plot for meta-analyses comparing the effect of GLP-1RAs with SGLT-2is in FBG.

**Figure. S2.** Forest plot for meta-analyses comparing the effect of GLP-1RAs with SGLT-2is in PBG.

**Figure. S3.** Forest plot for meta-analyses comparing the effect of semaglutide with SGLT-2is in bodyweight.

**Figure. S4.** Forest plot for meta-analyses comparing AEs of specific interest of GLP-1RAs with SGLT-2is.

**Table S1.** Search Strategy

| **Data source** | **Search terms** |
| --- | --- |
| **PubMed** | (“diabetes mellitus” OR “type 2 diabetes mellitus” OR T2DM OR weight OR BMI) AND (“SGLT 2 inhibitors” OR “sodium-glucose-co-transporter” OR “sodium glucose cotransporter” OR “sodium-glucose cotransporter” OR dapagliflozin OR empagliflozin OR canagliflozin OR luseogliflozin OR ipragliflozin OR tofogliflozin) AND (“Glucagon like peptide-1 receptor agonist” OR “GLP-1 receptor agonist” OR albiglutide OR naliglutide OR tanzeum OR dulaglutide OR trulicity OR exenatide OR bydureon OR Byetta OR liraglutide OR lixisenatide OR lyxumia OR semaglutide OR taspoglutid) AND metformin |
| **CENTRAL** | (“diabetes mellitus” OR “type 2 diabetes mellitus” OR T2DM OR weight OR BMI) AND (“SGLT 2 inhibitors” OR “sodium-glucose-co-transporter” OR “sodium glucose co transporter” OR “sodium-glucose cotransporter” OR dapagliflozin OR empagliflozin OR canagliflozin OR luseogliflozin OR ipragliflozin OR tofogliflozin) AND (“Glucagon like peptide-1 receptor agonist” or “GLP-1 receptor agonist” OR albiglutide OR naliglutide OR tanzeum OR dulaglutide OR trulicity OR exenatide OR bydureon OR Byetta OR liraglutide OR lixisenatide OR lyxumia OR semaglutide OR taspoglutid) AND Metformin in Title Abstract Keyword - (Word variations have been searched) |
| **Embase** | ('diabetes mellitus' OR 'type 2 diabetes mellitus' OR t2dm OR weight OR bmi) AND ('sodium-glucose-co-transporter' OR 'sodium glucose cotransporter' OR 'sodium-glucose cotransporter' OR dapagliflozin OR empagliflozin OR canagliflozin OR luseogliflozin OR ipragliflozin OR tofogliflozin) AND ('glucagon like peptide-1 receptor agonist' OR 'glp-1 receptor agonist' OR albiglutide OR naliglutide OR tanzeum OR dulaglutide OR trulicity OR exenatide OR bydureon OR byetta OR liraglutide OR lixisenatide OR lyxumia OR semaglutide OR taspoglutid) AND metformin AND [randomized controlled trial]/lim |
| **Web of Science** | (“diabetes mellitus” OR “type 2 diabetes mellitus” OR T2DM OR weight OR BMI) AND (“SGLT 2 inhibitors” OR “sodium-glucose-co-transporter” OR “sodium glucose cotransporter” OR “sodium-glucose cotransporter” OR dapagliflozin OR empagliflozin OR canagliflozin OR luseogliflozin OR ipragliflozin OR tofogliflozin) AND (“Glucagon like peptide-1 receptor agonist” OR “GLP-1 receptor agonist” OR albiglutide OR naliglutide OR tanzeum OR dulaglutide OR trulicity OR exenatide OR bydureon OR Byetta OR liraglutide OR lixisenatide OR lyxumia OR semaglutide OR taspoglutid) AND metformin |
| **Ovid** | #1 "randomized controlled trial".pt.  #2 (random$ or placebo$ or single blind$ or double blind$ or triple blind$).ti,ab.  #3 (retraction of publication or retracted publication).pt.  #4 or/#1-#3  #5 (animals not humans).sh.  #6 ((comment or editorial or meta-analysis or practice-guideline or review or letter or journal correspondence) not "randomized controlled trial").pt.  #7 (random sampl$ or random digit$ or random effect$ or random survey or random regression).ti,ab. not "randomized controlled trial".pt.  #8 #4 not (#5 or #6 or #7)  #9 (diabetes mellitus or type 2 diabetes mellitus or T2DM or diabet$ or obes$ or weight or BMI).tw.  #10 (SGLT 2 inhibitors or sodium-glucose-co-transporter or sodium glucose co transporter or sodium-glucose cotransporter or dapagliflozin or empagliflozin or canagliflozin or luseogliflozin or ipragliflozin or tofogliflozin).tw.  #11 (Glucagon like peptide-1 receptor agonist or GLP-1 receptor agonist or albiglutide or naliglutide or tanzeum or dulaglutide or trulicity or exenatide or bydureon or Byetta or liraglutide or lixisenatide or lyxumia or semaglutide or taspoglutid).tw.  #12 metformin.tw.  #13 #8 and #9 and #10 and #11 and #12 |
| **ClinicalTrials.gov** | (Tofogliflozin OR Empagliflozin OR dapagliflozin OR Canagliflozin OR Sotagliflozin OR luseogliflozin OR Ipragliflozin OR remogliflozin OR sergliflozin OR ertugliflozin) AND (albiglutide OR naliglutide OR dulaglutide OR exenatide OR liraglutide lixisenatide OR lyxumia OR semaglutide OR taspoglutide) |

**Table S2.** Quality assessment of randomized controlled trials included in the meta-analysis with the Cochrane Risk of Bias Assessment.

| Study | Random sequence  generation | Allocation  concealment | Blinding | Incomplete  outcome data | Selective  reporting | Other bias | Bias risk |
| --- | --- | --- | --- | --- | --- | --- | --- |
| Rodbard 2019 | Yes | Yes | No | Yes | No | Unclear | B |
| Lingvay 2019 | Yes | Yes | Yes | Yes | No | Unclear | A |
| Jabbour 2018 | Yes | Yes | Yes | Yes | No | Unclear | A |

A indicates low risk, B indicates unclear, C indicates high risk.

**Table S3. Sensitivity analyses for meta-analyses comparing the effect of GLP-1RAs with SGLT-2is in HbA1c. MD: mean diference, CI: confidence interval.**

|  | MD (95% CI) | *I^2^* |
| --- | --- | --- |
| Excluding trials with Rodbard 2019 | -0.27 (-0.36, -0.17) | 78% |
| Excluding trials with Lingvay 2019 | -0.22 (-0.30,-0.14) | 9% |
| Excluding trials with Jabbour 2018 | -0.32 (-0.40, -0.24) | 29% |

**Figure. S1.** Forest plot for meta-analyses comparing the effect of GLP-1RAs with SGLT-2is in FBG. CI: confidence interval, SD: standard deviation.


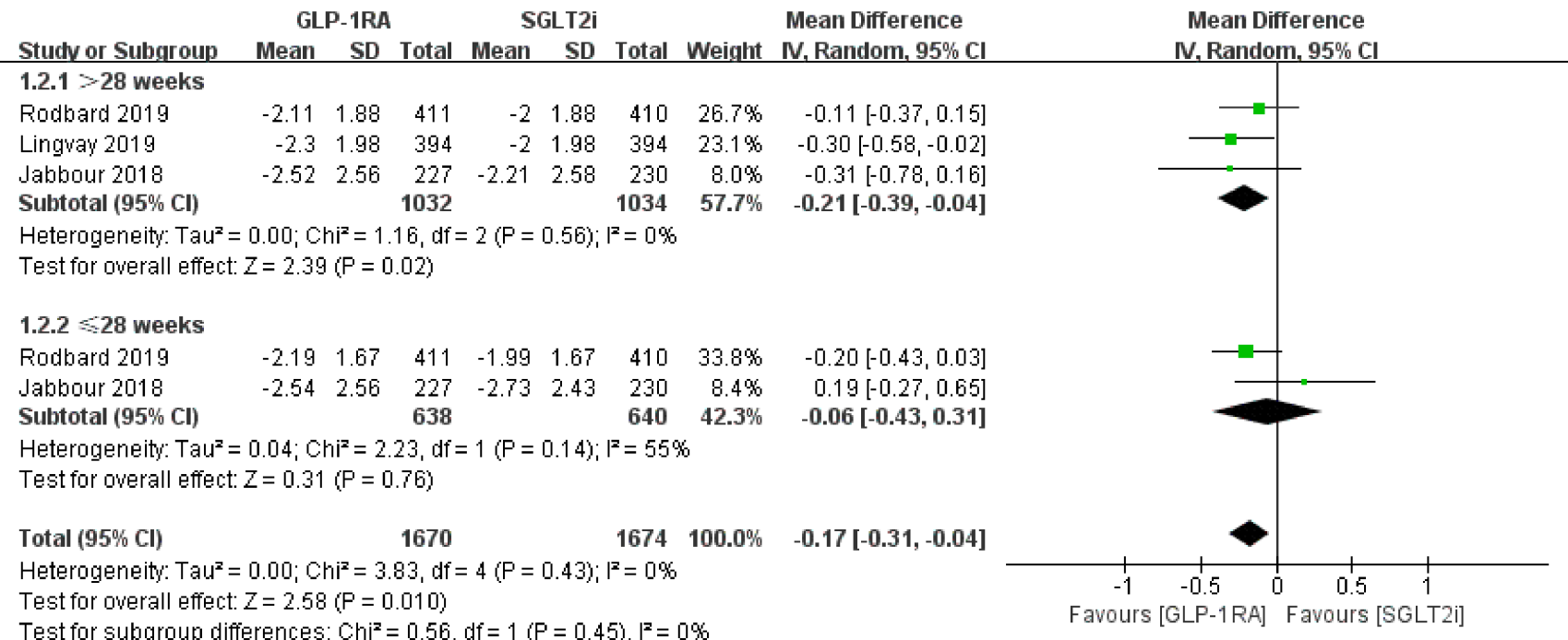


**Figure. S2.** Forest plot for meta-analyses comparing the effect of GLP-1RAs with SGLT-2is in PBG. CI: confidence interval, SD: standard deviation.





**Figure. S3.** Forest plot for meta-analyses comparing the effect of semaglutide with SGLT-2is in bodyweight. CI: confidence interval, SD: standard deviation.


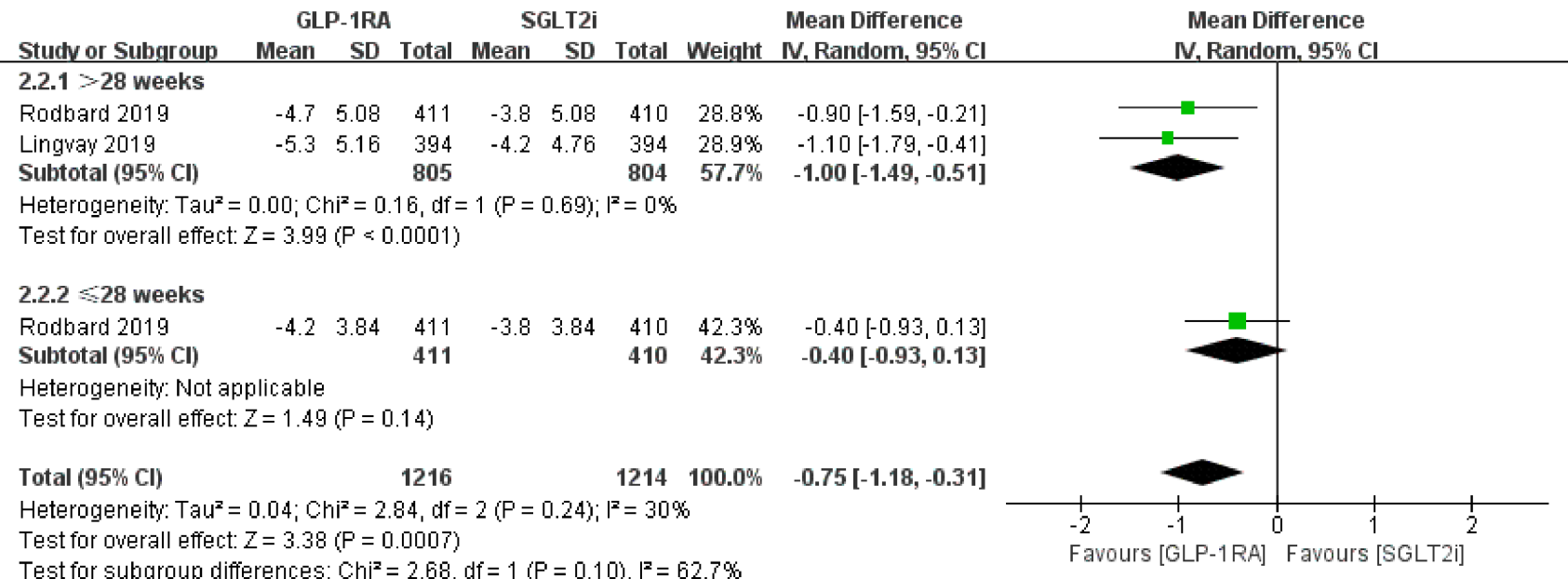


**Figure**

**. S4.** Forest plot for meta-analyses comparing AEs of specific interest of GLP-1RAs with SGLT-2is. (A) Hypoglycemia identified according to the ADA classifcation (<3·9 mmol/L [<70 mg/dL]). (B) Urinary tract infections. (C) Gastrointestinal events. CI: confidence interval.
